# The evolution of morphological development is congruent with the species phylogeny in the genus *Streptomyces*

**DOI:** 10.3389/fmicb.2023.1102250

**Published:** 2023-03-29

**Authors:** Min Wang, Cong-Jian Li, Zhen Zhang, Pan-Pan Li, Ling-Ling Yang, Xiao-Yang Zhi

**Affiliations:** ^1^Key Laboratory of Microbial Diversity in Southwest China of Ministry of Education, School of Life Sciences, Yunnan Institute of Microbiology, Yunnan University, Kunming, China; ^2^Zhaotong Health Vocational College, Zhaotong, China

**Keywords:** *Streptomyces*, morphological development, comparative phylogenetics, phylogenetic conflict, Robinson-Foulds distance

## Abstract

As the canonical model organism to dissect bacterial morphological development, *Streptomyces* species has attracted much attention from the microbiological society. However, the evolution of development-related genes in *Streptomyces* remains elusive. Here, we evaluated the distribution of development-related genes, thus indicating that the majority of these genes were ubiquitous in *Streptomyces* genomes. Furthermore, the phylogenetic topologies of related strict orthologous genes were compared to the species tree of *Streptomyces* from both concatenation and single-gene tree analyses. Meanwhile, the reconciled gene tree and normalization based on the number of parsimony-informative sites were also employed to reduce the impact of phylogenetic conflicts, which was induced by uncertainty in single-gene tree inference based merely on the sequence and the bias in the amount of phylogenetic information caused by variable numbers of parsimony-informative sites. We found that the development-related genes had higher congruence to the species tree than other strict orthologous genes. Considering that the development-related genes could also be tracked back to the common ancestor of *Streptomyces*, these results suggest that morphological development follows the same pattern as species divergence.

## Introduction

*Streptomyces* are Gram-positive and filamentous bacteria belonging to the phylum *Actinobacteria*. They have complex multicellular life cycles involving the transformation of a vegetative mycelium into the reproductive spores that are essential for their propagation (Kämpfer, 2015). Moreover, it is also the largest genus of prokaryotes, comprising 685 species with a validly published name (827 species including synonyms, data from LPSN)^1^ (Parte et al., 2020). The flourishing of species diversity is undoubtedly the result of its broad ecological adaptability (Andam et al., 2016; Li et al., 2019) and is also linked to its irreplaceable role in producing commercially and medically important bioactive substances (Anderson and Wellington, 2001). As we know, morphological differentiation and secondary metabolism are two physiologically coupled biological processes that strongly underpin the environmental adaptability of *Streptomyces* species (Sun et al., 2017; Xu et al., 2019). They have attracted the most interest and scrutiny of microbiological society.

The life cycle of *Streptomyces* species begins with the germination of a spore, followed by the formation of vegetative mycelia, including tip extension and the initiation of new branches (Flärdh and Buttner, 2009; van Dissel et al., 2014). Then, in response to nutrient depletion and other signals, the morphological differentiation and the production of secondary metabolites is initiated (Chater, 2006). Most of the genes involved in this intricate developmental process, e.g., *bld* genes related to the deficiency of aerial mycelium (Willey et al., 1991), *whi* genes related to spore formation (Soliveri et al., 2000), *ram* genes related to the hydrophobic protein synthesis (Capstick et al., 2007), *ssg* genes associated with the sporulation-specific cell division (Kormanec and Sevcikova, 2002), were considerably widespread in *Streptomyces*. Intriguingly, these two coupled biological processes were governed by complex regulatory networks (Bibb, 2005; Ohnishi et al., 2005). However, distinct from morphological differentiation, the secondary metabolism exhibits a high level of species specificity (Liu et al., 2013). Only some genes involved in regulation and signal transduction might be conserved among *Streptomyces* species (Bibb, 2005; Rodríguez et al., 2013).

Comparative genomics indicated that morphological development and its regulation in *Streptomyces* was probably achieved through the progressive acquisition of the laterally transferred DNA (Chater and Chandra, 2006). But most development-related genes may have been assembled in ancestors of *Streptomyces* species, at least before *Streptomyces* species branched off from the actinobacterial phylogenetic tree (Chandra and Chater, 2014). In this respect, *Streptomyces* differs from cyanobacteria, which also have the capacity for multicellular differentiation and a more complex early evolutionary history on the filamentous morphology (Sanchez-Baracaldo et al., 2005; Schirrmeister et al., 2011; Hammerschmidt et al., 2021). After that, the pan-genome analysis reaffirmed that many genes involved in stress response and morphological development were commonly expressed in *Streptomyces* species (Kim et al., 2015). Since 2013, fueled by rapid advances in high-throughput sequencing, the expansion of genome data of *Streptomyces*, especially the non-model streptomycetes (Lee et al., 2020), will aid in better comprehending the evolution of *Streptomyces* development. The relationship between gene phylogenies of specific core biosynthetic gene clusters and species phylogeny has been uncovered separately in a limited number of species (Joynt and Seipke, 2018; Waglechner et al., 2019; Navarro-Muñoz et al., 2020; Creamer et al., 2021). Nevertheless, the phylogenies of morphological development-related genes and their relationships with the phylogeny of species in *Streptomyces* remain elusive.

In this study, to address the above question, we performed an in-depth comparative phylogenetic investigation based on a large genome dataset of *Streptomyces*. The distributions of 65 development-related genes supported the previous conclusion that these genes were progressively assembled during the early evolution of *Streptomyces*, and some genes had been equipped in the genome of the common ancestor. Remarkably, the phylogenetic comparisons revealed that development-related genes had a higher degree of topological congruences to the species tree for both concatenated and single-gene trees. Whether by reconciling single-gene trees with the species tree using amalgamated likelihood estimation or by normalizing the topological distance of single-gene trees with a linear regression model, the development-related genes are always the gene sets that are considerably concordant with the species tree. These results demonstrated that the evolution of morphological development of *Streptomyces* kept in step with the speciation of this largest taxonomic taxon over the past 380 million years (McDonald and Currie, 2017).

## Materials and methods

### Genome and gene datasets preparation

The genome data (≥5 Mb) of type strains of 403 validly published *Streptomyces* species were collected from NCBI Assembly (344 genomes), GCM Type (43 genomes), and JGI IMG (16 genomes) databases (Supplementary Table 1). To reduce the redundancy of sequence data, the genome-pairwise average nucleotide identities (ANIs) were calculated by FastANI (Jain et al., 2018). And, if the genomes of two species shared an ANI value greater than 95%, which is the threshold for demarcating prokaryotic species (Chun et al., 2018), the genome of the species whose taxonomic name that had the priority of publication was retained (Li et al., 2022). Therefore, 388 genomes constituted a basic genome dataset (BGD) and were annotated using Prokka (version 1.12) with default parameters (Seemann, 2014). It needs to be stated that all subsequent phylogenetic analyses are based on the protein sequence. Firstly, 40 universal marker genes were extracted from BGD by SpecI (Mende et al., 2013), and the resultant gene datasets were denoted as UGs. Genomes with less than 38 extracted universal markers were further excluded from the BGD (Supplementary Table 2). Secondly, to collect the translated products of development-related genes in BGD, the reference protein sequences encoded by 65 development-related genes were retrieved from UniProt^2^ (Table 1) and used as queries to search against BGD by blastp (Altschul et al., 1990) with an *e*-value cut-off of 1 × 10^–5^. In each genome, all hits with identity ≥40% and coverage ≥50% were kept to constitute the original datasets for 65 development-related genes (denoted as DGs).

**TABLE 1:** The detailed information of development-related proteins in the genus *Streptomyces.*

Function category	Name	Length (aa)	Entry (UniProt)	Description	Organism	References
A-factor	AdpA	405	Q9S166	AdpA, A-factor-responsive transcriptional activator	*S. griseus*	Ohnishi et al., 1999
	ArpA	276	Q9ZN78	A-factor receptor protein	*S. griseus*	Onaka et al., 1995
	AfsA	301	P18394	2-oxo-3-(phosphooxy)propyl 3-oxoalkanoate synthase	*S. griseus*	Horinouchi et al., 1989
	BprA	282	B1VN94	(4-alkanoyl-5-oxo-2,5-dihydrofuran-3-yl)methyl phosphate reductase	*S. griseus*	Ohnishi et al., 2008
Aerial hypha formation	SigJ	198	Q9K2W6	ECF subfamily RNA polymerase sigma factor	*S. coelicolor*	Mazurakova et al., 2006
	SigN	278	Q9ADM4	Putative RNA polymerase sigma factor	*S. coelicolor*	Dalton et al., 2007
	BldA	402	Q93JF9	BldA-regulated nucleotide binding protein	*S. coelicolor*	Bentley et al., 2002
	BldB	98	Q7AKF6	Regulator, BldB	*S. coelicolor*	Bentley et al., 2002; Eccleston et al., 2006
	BldC	68	Q9RKJ5	Developmental transcriptional regulator BldC (MerR family)	*S. coelicolor*	Hunt et al., 2005
	BldD	167	Q7AKQ8	DNA-binding protein	*S. coelicolor*	Elliot et al., 1998
	BldG	113	Q9WVX8	Anti-sigma factor antagonist (Anti-sigma-B factor antagonist)/Anti-sigma-B factor antagonist	*S. coelicolor*	Bignell et al., 2003
	BldKA	343	Q93IU3	ABC transporter integral membrane protein BldKA	*S. coelicolor*	Bentley et al., 2002
	BldKB	600	Q93IU2	ABC transporter lipoprotein BldKB	*S. coelicolor*	Chávez et al., 2011
	BldKC	325	Q93IU1	ABC transporter integral membrane protein BldKC	*S. coelicolor*	Bentley et al., 2002
	BldKD	353	Q93IU0	ABC transporter intracellular ATPase subUT BldKD	*S. coelicolor*	Bentley et al., 2002
	BldKE	381	Q8CJS2	Peptide transport system ATP-binding subUT	*S. coelicolor*	Bentley et al., 2002
	BldM	203	Q7AKI8	Putative two-component regulator (isolation response regulatory proteins)	*S. coelicolor*	Bentley et al., 2002
	BldN	177	Q9WX11	RNA polymerase sigma factor	*S. coelicolor*	Bentley et al., 2002
	ClpP1	219	Q9F315	ATP-dependent Clp protease proteolytic subUT 1	*S. coelicolor*	de Crecy-Lagard et al., 1999
	ClpX	428	Q9F316	ATP-dependent Clp protease ATP-binding subUT ClpX	*S. coelicolor*	Bentley et al., 2002
	SigH	361	Q9RIT0	RNA polymerase sigma factor	*S. coelicolor*	Potúèková et al., 1995
	Sti1	144	P61152	Subtilase-type protease inhibitor	*S. coelicolor*	Kim et al., 2005
Cell division	CrgA	84	Q9XA10	Cell division protein CrgA	*S. coelicolor*	Bentley et al., 2002; Del Sol et al., 2003
	DpsA	187	Q9R408	DNA-binding protein from starved cells	*S. coelicolor*	Facey et al., 2009
	DpsB	425	O86816	DNA-binding protein from starved cells	*S. coelicolor*	Facey et al., 2009
	DpsC	353	Q9K3L0	DNA-binding protein from starved cells	*S. coelicolor*	Facey et al., 2009
	FtsI	651	Q9Z5V7	FtsI	*S. coelicolor*	Bennett et al., 2009
	FtsK	917	O86810	DNA translocase FtsK	*S. coelicolor*	Bentley et al., 2002
	FtsQ	264	P45518	Cell division protein FtsQ	*S. coelicolor*	McCormick and Losick, 1996
	FtsW	456	Q9ZBA6	Peptidoglycan glycosyltransferase	*S. coelicolor*	Mistry et al., 2008
	MreB	361	Q9L1G6	Cell shape-determining protein MreB	*S. coelicolor*	Heichlinger et al., 2011
	ParB1	381	Q9S6U1	Putative plasmid partitioning protein, ParB2	*S. coelicolor*	Kim et al., 2000; Bentley et al., 2002
	SffA	596	Q9RKX5	Putative FtsK/SpoIIIE family protein	*S.coelicolor*	Ausmees et al., 2007
	Smc	1,186	Q9ZBQ2	Chromosome associated protein	*S. coelicolor*	Bentley et al., 2002; Dedrick et al., 2009
	SmeA	64	Q9RKX4	Putative small membrane protein	*S.coelicolor*	Ausmees et al., 2007
	DivIVA	398	Q9S2X4	Cell division protein DivIVA	*S. coelicolor*	Xu et al., 2008
	FtsZ	399	P45500	Cell division protein FtsZ	*S. coelicolor*	McCormick et al., 1994
	ParA	310	Q8VWE4	Putative partitioning protein ParA	*S. coelicolor*	Bentley et al., 2002; Ditkowski et al., 2010
	ParJ	333	Q9ADA5	Filament polymerization regulator ParJ	*S. coelicolor*	Xu et al., 2020
Hydrophobin	ChpA	252	Q8CJY7	Chaplin-A	*S. coelicolor*	Bentley et al., 2002
	ChpB	237	Q9X7U2	Chaplin-B	*S. coelicolor*	Bentley et al., 2002
	ChpC	259	Q9AD93	Chaplin-C	*S. coelicolor*	Bentley et al., 2002
	ChpD	75	Q9L1J9	Chaplin-D	*S. coelicolor*	Bentley et al., 2002
	ChpE	82	Q9X9Z2	Chaplin-E	*S. coelicolor*	Bentley et al., 2002
	ChpF	88	Q9KYG7	Chaplin-F	*S. coelicolor*	Bentley et al., 2002
	ChpG	90	Q9KYH3	Chaplin-G	*S. coelicolor*	Bentley et al., 2002
	ChpH	77	Q9AD92	Chaplin-H	*S. coelicolor*	Bentley et al., 2002
	RamA	635	O88039	ABC transporter ATP-binding protein	*S. coelicolor*	Ma and Kendall, 1994; Bentley et al., 2002
	RamB	608	Q7AKE5	ABC transporter ATP-binding protein	*S. coelicolor*	Ma and Kendall, 1994; Bentley et al., 2002
	RamC	930	O88037	Probable SapB synthase	*S. coelicolor*	Bentley et al., 2002
	RamR	202	Q7AKE4	Two-component system response regulator/rapid aerial mycelium regulator	*S. coelicolor*	Ma and Kendall, 1994; Bentley et al., 2002
	RamS	42	O88038	Lanthionine-containing peptide SapB precursor RamS	*S. coelicolor*	Bentley et al., 2002
Sporulation	SigF	287	P37971	RNA polymerase sigma-F factor	*S. coelicolor*	Kim et al., 2008; Ditkowski et al., 2010
	SsfR	326	Q9L688	Putative transcriptional regulator	*S. griseus*	Jiang and Kendrick, 2000
	SsgA	136	Q9X9U2	Regulator (Sporulation/cell division regulator)	*S. coelicolor*	Bentley et al., 2002
	SsgB	137	Q9L268	Sporulation-specific cell division protein SsgB	*S. coelicolor*	Xu et al., 2009
	SsgR	241	Q9X9U3	Transcriptional regulator	*S. coelicolor*	Bentley et al., 2002
	WhiA	328	Q9Z515	Probable cell division protein WhiA (hypothetical protein)	*S. coelicolor*	Aínsa et al., 2000
	WhiB	87	Q7AKN0	Transcriptional regulator WhiB	*S. coelicolor*	Davis and Chater, 1992
	WhiD	112	Q7AKI9	Transcriptional regulator WhiD	*S. coelicolor*	Soliveri et al., 2000
	WhiE	627	P42534	Putative polyketide hydroxylase	*S. coelicolor*	Komaki et al., 2015
	WhiG	280	P17211	RNA polymerase sigma factor WhiG	*S. coelicolor*	Chater et al., 1989
	WhiH	295	Q7AKF5	Sporulation transcription factor, WhiH	*S. coelicolor*	Genay et al., 2007
	WhiI	220	O69859	Two-component regulator	*S. coelicolor*	Ainsa et al., 1999; Bentley et al., 2002
	WhiJ	283	Q9F3F8	Transcriptional regulator WhiJ	*S. coelicolor*	Aínsa et al., 2010

### Random genome datasets generation

Because of the large number of genomes in the basic genome dataset, the current orthology inference method cannot obtain sufficient strict orthogroups [theoretically refer to a gene family including genes that are orthologous to each other and do not involve pairs of inparalogs (Boeckmann et al., 2011); technically refer to a gene family including genes that are not only homologous to each other but also single copy in the source genomes]. Therefore, we used a random resampling strategy similar to previous works (Canback et al., 2002; Zhi et al., 2014) to decrease the number of genomes and to cover as much species diversity presented in BGD as possible. Briefly, a reference species tree (denoted as RST) was first reconstructed based on a concatenated alignment of UGs, using IQ-tree (version 2.1.2) (Nguyen et al., 2015). Due to the asymmetry of RST in the number of species on the deep branches, it was re-rooted to enable the numbers of species contained in the two deepest branches to be close. And then, based on re-rooted RST, all species were divided into groups based on different clades (Supplementary Table 3). The corresponding genomes were randomly selected in terms of the species constitution of various clades in RST. Finally, random sampling was repeated to generate 100 random genome datasets (denoted as RGDs, Supplementary Table 4).

### Phylogenetic tree inference

All RGDs were individually subjected to orthology inference using OrthoFinder (version 2.5.2) (Emms and Kelly, 2015, 2019). The orthogroups with precisely one gene from each genome were regarded as the strict orthogroups (sOGs) and were aligned separately by ClustalO (version 1.2.3) (Sievers et al., 2011). Then, all alignments of sOGs were concatenated, and the ambiguous sites were trimmed by trimAl (version 1.4) (Capella-Gutierrez et al., 2009) with the “automated” option. A species tree based on supermatrix (denoted as ST1) was inferred using IQ-tree, based on the best model with the following options: “–m TEST –B 1000 –T AUTO.” To alleviate the bias induced by a single method, an additional species tree based on supertree (denoted as ST2) under the multispecies coalescent model was also built using ASTRAL (version 5.7.4) (Mirarab et al., 2014). The single-gene maximum likelihood (ML) trees used in the supertree approach were inferred using IQ-tree with options: “-m TEST –score-diff ALL –B 1000 –wbtl –T AUTO.”

Besides ST1 and ST2, other two concatenated ML trees were reconstructed based on UGs and DGs, respectively. Because the universal marker gene had at most one sequence in each genome, UGs can be treated like sOGs, and their concatenated tree (denoted as UT) could be built using the same method. However, unlike UG, the development-related gene sequences belonging to one RGD were first extracted from the original DG (based on BGD) and then subjected to the orthology assessment by UPhO (Ballesteros and Hormiga, 2016). To a DG family, its subfamilies that formed the largest monophyletic subtrees in the tree of the whole family were recognized as strict orthogroups and used to build the concatenated ML tree (denoted as DT). In summary, for each RGD, two species trees (ST1 and ST2), two concatenated ML trees (UT and DT), and a batch of single-gene trees (genes in sOGs and DGs) were reconstructed as well.

### Phylogenetic topology comparison

**FIGURE 1:**
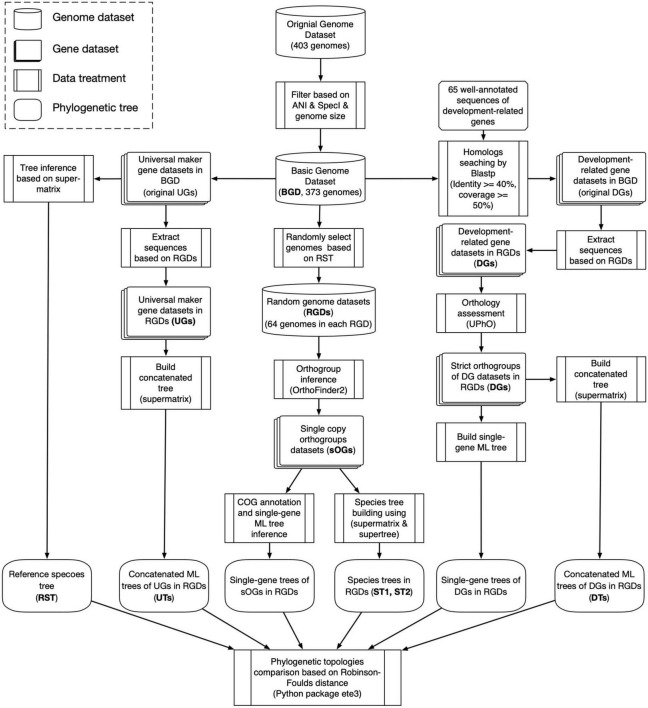
Flowchart of data analysis in this study.

The Robinson-Foulds (RF) distance (Briand et al., 2020; Hayati and Chindelevitch, 2020) was employed to quantify the difference in phylogenetic topologies by using the Python package ete3 (version 3.1.2). The species trees were compared with concatenated ML trees and single-gene trees. However, because multichotomous branching profoundly affects the RF distance calculation, the single-gene trees with multichotomous branches involving ≥50% of species were excluded from the topological comparison. To examine the difference in phylogenetic topologies among various functional groups, the Clusters of Orthologous Genes (COG) annotation was performed on all sOGs using the online version of eggNOG (version 5.0) (Huerta-Cepas et al., 2019). The single-gene trees of sOGs could be divided into subgroups according to the COG categories (Galperin et al., 2021), and their RF distances to species trees (ST1 and ST2) were analyzed separately. The whole data analysis procedure in this work is illustrated schematically in Figure 1.

The underpinning evolutionary processes causing phylogenetic conflict are diverse (Schrempf and Szöllősi, 2020). Besides biological factors like gene duplication and loss, and horizontal gene transfer, a wide variety of analytical factors, e.g., the tree reconstruction based on sequence alone (Szöllősi et al., 2013) and the limited amount of phylogenetic information (Steel, 2005), can lead to overestimating the phylogenetic conflict. Here, to reduce the discordance between gene trees inferred based on sequence alone, single-gene trees were reconciled using amalgamated likelihood estimation (ALE version 0.4) (Szöllősi et al., 2013, 2015). Briefly, the ultrafast bootstrap tree distributions generated by IQ-tree were converted into ALE objects using ALEobserve. And then, each ALE object was reconciled using ALEml_undated with the species tree.

Additionally, to reduce the gene tree incongruence due to differences in the number of parsimony-informative sites (PIS), the RF distance between the species tree and single-gene trees were normalized using a linear model between the PIS numbers and the actual RF distances. All RF distance values were sorted by corresponding PIS numbers ascendingly and divided into equal-sized bins (each bin contained 1,000 RF values). In each bin, the top 5% of RF values (the 5th percentile of RF values) representing “good” single-gene trees were selected for regression analysis with the relevant PIS numbers. A linear model was used to fit a line (RF = alog_10_N_PIS_+b, N_PIS_: the number of PIS) using least squares fitting. Then, all original RF values were normalized using the formula ${\text{RF}}^{{}^{\prime }}=\frac{\text{RF}}{\text{a}\,\log_{10}{\text{N}}_{\text{PIS}}+\text{b}}.$

## Results

### The morphological development predated the species divergence of *Streptomyces*

Based on 40 universal markers, a reference species tree (RST) of 373 *Streptomyces* genomes (basic genome datasets, BGD) was reconstructed. According to the evolutionary relationships reflected in RST, 373 *Streptomyces* species were segregated into eight major clades labeled as C1–C8 (Supplementary Figure 1). The species numbers of these clades were 1 (C1), 2 (C2), 18 (C3), 159 (C4), 36 (C5), 56 (C6), 15 (C7), and 86 (C8), respectively (Supplementary Table 3). Namely, the two deepest internal branches of the re-rooted RST possessed four descendant clades: C1–C4 (180 species) and C5–C8 (193 species). Parts of *Streptomyces* genomes were randomly selected according to the proportion of species number in eight clades, and their genomes constituted a small random genome dataset (RGD) containing 64 species (Supplementary Table 4). It’s worth noting that the number of species in each major clade is varied; hence the probabilities of 373 species being selected are also different. The highest proportion of overlapped species between RGDs was less than 37.2%. And each species was re-sampled an average of 17.3 times (standard deviation, sd: 6.2).

**FIGURE 2:**
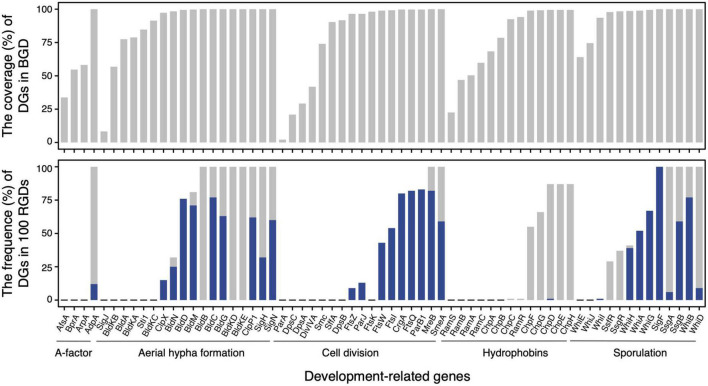
The distributions of development-related genes in the basic genome dataset **(upper panel)** and 100 random genome datasets **(lower panel)**. In the lower panel, the frequencies of genome dataset (DG) as core gene family (having homolog in each genome) and strict orthogroup (subfamily assessed by UPhO) are visualized as gray and blue bars, respectively.

In terms of the distribution of homologous sequences, as expected, 44 of 65 development-related genes were identified over 90% of genomes in BGD (Figure 2, upper panel). Those genes ought to play crucial roles in the development of *Streptomyces*. For instance, AdpA, an essential early regulator of the development (Horinouchi, 2007), and MreB, involved in the spore cell wall assembly (Heichlinger et al., 2011) could be identified in all *Streptomyces* genomes. Nevertheless, nearly a third of the development-related genes showed varying degrees of loss. Most of them were either unnecessary for morphological development or could be replaced by alternative genes. For example, the narrow phylogenetic distribution of the SapB biosynthesis gene cluster, including the *ramCSAB* operon and *ramR* (a response regulator gene that controls the activation of the convergently transcribed *ramCSAB* operon), might relate to a SapB-independent pathway that is mediated by the chaplins (Nguyen et al., 2002; O’Connor et al., 2002). These results are consistent with the previous report (Chandra and Chater, 2014; Kim et al., 2015). However, the distribution of homologous sequences cannot reflect evolutionary events such as horizontal gene transfer and gene duplication. In other words, a gene of late origin can exhibit a wider phylogenetic distribution through HGT. Therefore, we evaluated the orthologies of these DGs and further investigated the distribution of orthologous DGs.

However, because it is difficult to obtain a reliable and stable phylogenetic result for the large sequence dataset, evaluating the orthology of DG directly based on BGD is not feasible. Therefore, the assessment of orthology was carried out based on RGDs in this work. With the reduction of the BGD to an RGD comprising 64 genomes, the development-related protein sequences only associated with focal RGD were extracted from the original DGs. In focal RGD, the development-related protein family distributed in all 64 genomes (number of sequences ≥64) was regarded as a core protein family and subjected to the assessment of orthology. The subfamilies of the core protein family corresponding to strict orthogroups were finally retained. Due to the high similarity between some query sequences (e.g., the similarities among SigH, SigF, and SigN were more than 70%), the identical strict orthogroup might be extracted from different DGs. In this case, only the strict orthogroup shared the highest similarity to the corresponding reference was extracted for further investigation. In addition, five protein families (ClpP1, SigN, SigF, WhiD, and SmeA) could be decomposed into two subfamilies as strict orthogroups and involved in 33 RGDs. Particularly, this situation occurs mainly in two protein families: ClpP1 (19 RGDs) and SigN (10 RGDs). These 33 RGDs included one and only one development-related gene that has this situation except for RGD064 and RGD090. All these strict orthogroups were retained for subsequent analysis.

The distributions of orthologous DGs in BGD were exhibited through the frequencies of DGs as core protein families (gray bars) and as strict orthogroups (blue bars) in 100 RGDs (Figure 2, lower panel). A total of 29 DGs involved in aerial hyphae and mature spore formation, cell division, and hydrophobin synthesis were recognized as core protein families in more than 50 % of RGDs. And 17 of 29 could extract strict orthogroups. These protein families from which strict orthogroups can be extracted tend to be more conserved than others. Notably, three protein families (BldB, BldKE, and BldKD) failed to extract the strict orthogroups, due to the high identity between sequences (resulting in a multifurcating tree). In addition, even though the strict orthogroups could be retrieved, the trimmed alignments were so short and conservative that the comparable trees of BldC, BldM, SsgB, WhiB, CrgA, and SmeA were unavailable. Besides the genome incompleteness and the species specificity of certain proteins, the progressive acquisition of these genes in the early ancestors of *Streptomyces* might also account for the differences in protein family distribution. Based on the larger genome datasets and relatively comprehensive protein collection, these results demonstrated that development-related genes had already been equipped in the genome of the common ancestor of *Streptomyces* and underwent a vertical evolutionary history, confirming that the origin of development should occur before the flourishing of its species diversity.

### The concatenated DGs phylogeny has a higher degree of congruence with the species tree

**FIGURE 3:**
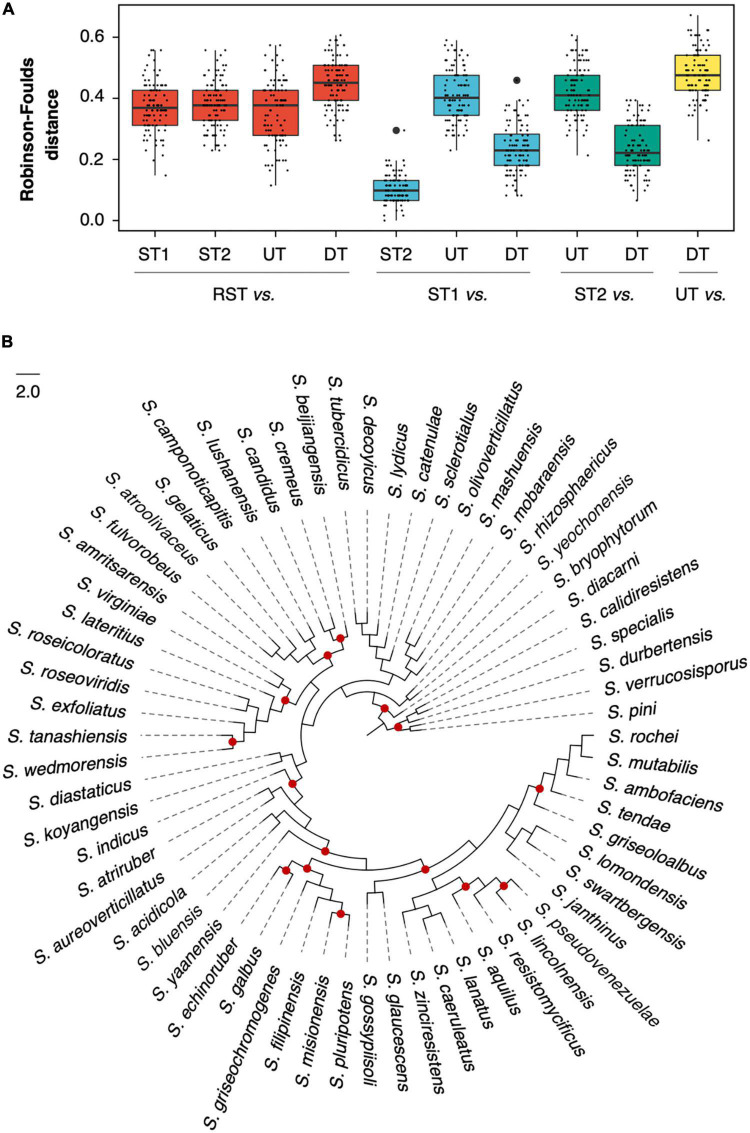
Topological comparison among species trees and concatenated maximum likelihood (ML) trees. **(A)** Robinson-Foulds distance between two compared trees in one of 100 random genome datasets (RGDs); **(B)** the species tree of RGD008 based on the supermatrix approach. The red dots represent where the phylogenetic conflict between DT and ST1 occurred.

In each RGD, two species trees based on supermatrix (ST1) and supertree (ST2) approaches and two concatenated ML trees, DT (based on DGs) and UT (based on UGs), were reconstructed. And a batch of single-gene trees was also inferred for strict orthogroups (sOGs) (496 60.8 trees) and DGs (10 2.6 trees). The Robinson-Foulds (RF) distance was implemented to evaluate the topological differences. Noticeably, when RST was compared to ST1, ST2, DT, and UT; it was required to be pruned to fit the different comparison objects. As shown in Figure 3A, the RF distances from UT to RST (mean sd: 0.36 0.1) were not significantly different from the distances from ST1 to RST (0.37 0.08) (Student’s *t*-test, *p* = 0.167). Compared to RST, ST1 differed in both the underlying gene dataset and the number of species involved; but UT only differed in the number of species involved. Therefore, it indicated that the random resampling strategy could achieve the goal of maintaining topological stability when reducing the number of species involved. Nevertheless, in addition to the underlying gene dataset and the number of species involved, the species tree-building approach of ST2 was also different. That might cause a significant difference between the distances from ST2 to RST and that from UT to RST (*t*-test, *p* = 0.048). The RF distances from DT to RST were significantly higher than that from ST1, ST2, and UT to RST (*t*-test, all *p*-values < 10^–5^).

When ST1 was used as a reference, the RF distances from DT to ST1 decreased dramatically (from 0.45 0.08 to 0.23 0.08) and were even significantly lower than that from UT to ST1 (*t*-test, *p* < 10^–5^). For example, Figure 3B illustrates the degree of congruence between DT and ST1, which were generated from dataset RGD008 (RF = 0.23). Given the negligible difference between ST1 and ST2 (their average RF distance was only 0.10 0.05), the distances of DT decreased similarly when ST2 was used as a reference. For further understanding of the distances from DT to ST1, sOGs were divided according to COG functional categories and then used to infer concatenated ML trees for each COG category. The average RF distances from COG categories to ST1 ranged from 0.11 (COG Others, including orthogroups that could not be assigned to COGs or whose COG category appeared once in 100 RGDs) to 0.33 (COG P) (Supplementary Table 5). Interestingly, DT had a lower RF distance to ST1 than half of the COG categories. Concerning concatenation analysis, the phylogenies of concatenated DGs had a higher degree of congruence with the specie trees of the genus *Streptomyces*.

### The single-gene phylogenies of DGs have no significant difference from that of sOGs on phylogenetic conflict

**FIGURE 4:**
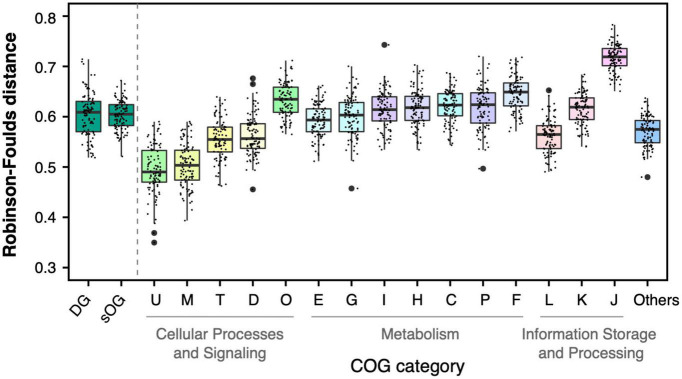
Topological comparison between single-gene trees and species tree based on supermatrix (ST1). Each single data point represented a mean value of Robison-Foulds distances between single-gene trees in the corresponding dataset [e.g., genome dataset (DG)] to ST1 in one of 100 random genome datasets (RGDs).

The concatenation approach integrating phylogenetic information from multiple gene loci suppresses the phylogenetic heterogeneity between different genes (Pease et al., 2016; Wu et al., 2018; Jiang et al., 2020). To investigate the phylogenetic conflicts of single genes with the species tree, all single-gene trees in different gene datasets were individually compared with the species tree (Figure 4 and Supplementary Figure 2, detailed data see Supplementary Table 5). For each gene dataset, the mean value of RF distances from single-gene trees to the species tree was calculated and used to measure the topological difference between the whole gene dataset and the species tree. The distribution of mean values of RF distances from sOGs to ST1 demonstrated that the single-gene tree had a higher degree of incongruence to the species tree than the concatenated tree, as expected.

The mean RF distances from DGs to ST1 (0.60 0.04) were not significantly different from the mean RF distances from sOGs to ST1 (0.60 0.03) (*t*-test, *p* = 0.9). And it is consistent with the results of concatenation analysis, DGs had lower RF distance to ST1 than parts of COG categories (e.g., COGs F and J). sOGs belonging to different COG categories showed different degrees of topological incongruence with species trees. For instance, COG M (cell wall/membrane/envelope biogenesis) and COG U (intracellular trafficking, secretion, and vesicular transport) had relatively lower mean RF distances to the species tree. And, at the higher level of functional classification, the single-gene trees of COGs related to cellular processes and signaling were closer to the species tree than those of other COGs involved in metabolism and information storage and processing. Astonishingly, COG J (translation, ribosomal structure, and biogenesis), including all ribosomal proteins usually used in phylogenomic analysis exhibited the highest degree of incongruence with the species tree. However, the concatenated tree of COG J had only 0.26 RF distance on average to ST1 (Supplementary Table 5). This suggested that the concatenation would exert a complex effect on species tree reconstruction.

**FIGURE 5:**
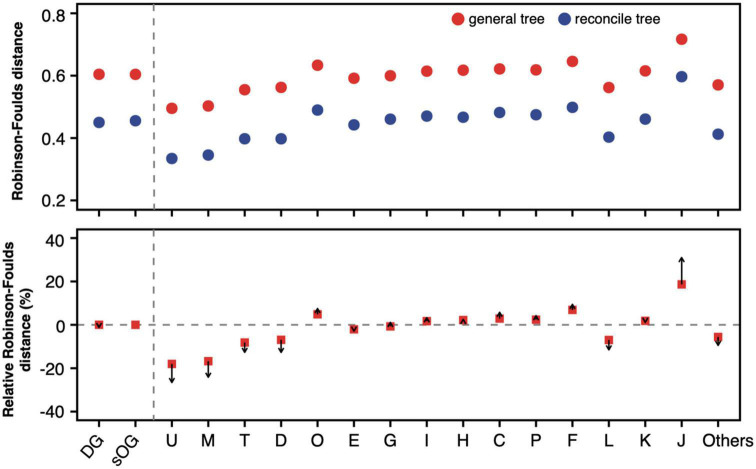
Robinson-Foulds distance of general gene tree and reconciled gene tree to supermatrix (ST1). For a gene dataset, 100 mean values of Robinson-Foulds (RF) distances from 100 random genome datasets (RGDs) were averaged and shown in the **(upper panel)**. Red solid circles represent the average RF distance of general gene trees to ST1, and solid blue circles represent the average RF distance of reconciled gene trees to ST1. The relative RF distance was calculated as formula, $\frac{{\bar{\text{RF}}}_{{\text{t}}_{\text{J}}\to \mathrm{ST1}}-{\bar{\text{RF}}}_{{\text{t}}_{\text{sOG}}\to \mathrm{ST1}}}{{\bar{\text{RF}}}_{\text{sOG}\to \mathrm{ST1}}}$, where t_J_ is general gene tree of the gene in Clusters of Orthologous Genes (COG) J. In the **(lower panel)**, arrows start from the relative RF distance of general gene trees and point to the relative RF distance of reconciled gene trees.

The phylogenetic inference of a single gene based on sequence alone often lacks enough information to confidently support one gene tree topology. This may be one of the major reasons why the single gene has a higher degree of topological incongruence than the concatenated alignment. By reconciling the gene tree with a putative species tree based on the joint likelihood, its incongruence with the species tree was substantially reduced (Figure 5 and Supplementary Figure 3). However, the mean RF distances of reconciled gene trees are still higher than those of the corresponding concatenation trees. Interestingly, although the RF distances were decreased after the gene trees were reconciled, the magnitudes of the RF distance reduction of various gene datasets relative to sOGs were discrepant. Generally, gene datasets whose single-gene trees were closer to the species tree, such as COGs M and U, had more magnitudes of RF distance reduction. In contrast, gene datasets whose single-gene trees were more distant from the species tree, such as COG J, had fewer magnitudes of RF distance reduction.

### DGs exhibited the highest topological concordance with the species tree based on the normalized RF distance

**FIGURE 6:**
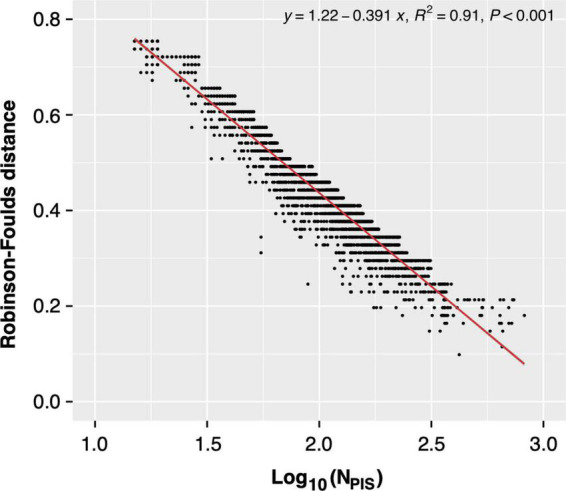
Regression analysis between Robinson-Foulds (RF) distance [between the single-gene tree and supermatrix (ST1)] and the number of parsimony-informative sites.

As we know, the number of information sites used for phylogenetics profoundly impacted the topology of the resultant tree. Therefore, the limited number of parsimony-informative sites might contribute to the topological incongruence between the single gene tree and the species tree. Meanwhile, we noted that the numbers of parsimony-informative sites of proteins seem to be negatively correlated to their RF distances (Pearson’s *r* = -0.51, *p* < 0.01, Supplementary Table 5). Therefore, to determine the correlation between the number of parsimony-informative sites and the topological incongruence, all single genes in different gene datasets were divided into bins according to the number of parsimony-informative sites. The RF distance values in each bin were sorted ascendingly, and the top 5% of RF values representing ones with “good” single-gene phylogeny (similar to species tree) were used for the regression analysis (Figure 6 and Supplementary Figure 4).

**FIGURE 7:**
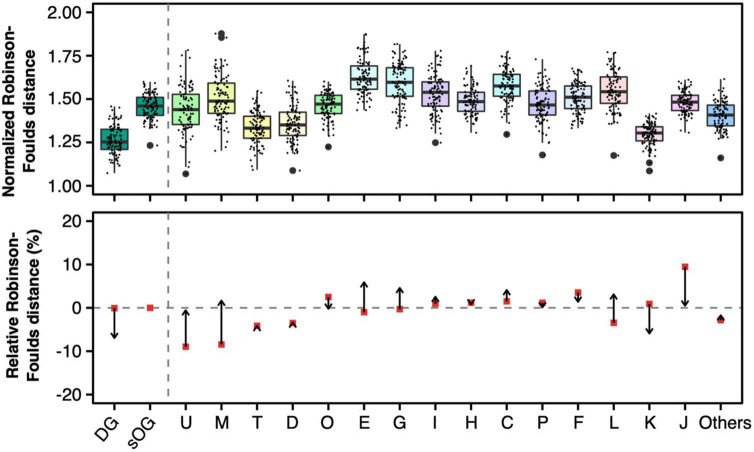
Normalized Robison-Foulds distances **(upper panel)** and relative normalized Robison-Foulds distances (**lower panel)** between single-gene trees and supermatrix (ST1). Like the lower panel of Figure 4, the arrows start from the relative Robison-Foulds (RF) distances of general gene trees and point to the relative RF distances after normalization based on the number of parsimony-informative sites.

The actual RF distances were normalized based on the linear regression model (Figure 7 and Supplementary Figure 5). However, the normalized RF distance scale changed, which made the direct comparison to the original RF distance inapplicable. So, just as reconciled gene tree comparison, relative RF distance was used to present the variation of RF distance caused by the normalization based on the number of parsimony-informative sites. The relative mean RF distance from single-gene trees of COG J to ST1 decreased from 18.6 to 1.2%; on the contrary, the relative mean RF distance from single-gene trees of COG L to ST1 increased from –6.9 to 6.0%. Generally, the regression model decreased the RF distances of genes with more parsimony-informative sites (such as COGs U, M, E, G, C, and L). In contrast, the RF distances of genes with a lower number of parsimony-informative sites were increased (such as COGs J, K, and DGs). Remarkably, the RF distances of DGs had dramatically changed relative to sOGs same as COG J (decreased from <0.1 to –13.2%), and became the lowest ones. Taken together, these results indicated that development-related genes exhibited more congruent single-gene phylogenies to the species tree versus strict orthogroups, especially when technical factors like the limitation of parsimony-informative sites are excluded.

## Discussion

The development of *Streptomyces*, including both morphological and physiological differentiation, was controlled by the same complex regulatory system and endowed streptomycetes with the ability to cope with complex living environments. Based on a large genome dataset, we found that development-related genes present differential distribution patterns in *Streptomyces* genomes. Parts of development-related genes had been equipped in the genome of the common ancestor of *Streptomyces* bacteria. Although it remains unknown whether these are sufficient to support the morphological development of the common ancestor of *Streptomyces*, the origin of these genes can be traced back to the early stages of *Streptomyces* speciation or even earlier. The phylogenetic distribution of homologous genes is an essential tool for studying the origin of genes and relevant biological functions, relying on the assumption that ancient genes often have a broader phylogenetic distribution. However, the natural evolutionary process is not entirely immune to confounding factors, such as horizontal gene transfer, which inevitably complicate the evolution of the gene family. In this study, the orthologies of development-related genes were also considered besides their phylogenetic distribution.

Furthermore, we compared the phylogenetic topologies of related genes to the species tree on both concatenation and single-gene levels. We found that the evolution of development-related genes had higher congruence to the species tree. Especially when the analytical factors, such as tree reconstruction based on sequence alone and the limited amount of phylogenetic information, were excluded, the development-related genes exhibited the lowest degree of topological incongruence with the species tree. This is consistent with the above conclusion that parts of development-related genes have been assembled in the genome of the comment ancestor of *Streptomyces*. Consequently, the subsequent evolution of these genes should be synchronized with the history of species differentiation. Or in other words, the development-related genes documented the evolutionary history of *Streptomyces*. Meanwhile, it must be noted that the phylogenies of some development-related genes originating earlier in *Streptomyces* were relatively complex. These genes were either not conservative enough, or their genuine orthologies were obscured by other evolutionary events like lateral gene transfer, gene duplication, and loss.

## Data availability statement

Publicly available datasets were analyzed in this study. This data can be found here: doi: 10.5061/dryad.00000005d.

## Author contributions

MW and C-JL: initial drafting, data analysis, figures generation, and discussion. ZZ and P-PL: raw data processing and discussion. L-LY: discussion and draft correction. X-YZ: project coordination, discussion, and draft correction. All authors contributed to the article and approved the submitted version.

## Footnotes

1
https://lpsn.dsmz.de/genus/streptomyces

2
https://www.uniprot.org
