# Supplementary material for: The evolution of morphological development is congruent with the species phylogeny in the genus Streptomyces
Source: Front Microbiol. 2023 Mar 29;14:1102250. doi: 10.3389/fmicb.2023.1102250 (PMC10090380; doi:10.3389/fmicb.2023.1102250)
Supplement: Supplementary file 2 [file Data_Sheet_1.docx]

**Supplementary materials**

**The evolution of morphological development is congruent with the species phylogeny in the genus *Streptomyces***

Min Wang^1,2,†^, Cong-Jian Li^1,†^, Zhen Zhang^1^, Pan-Pan Li^1^, Ling-Ling Yang^1^ and Xiao-Yang Zhi^1,*^

^1^ Key Laboratory of Microbial Diversity in Southwest China of Ministry of Education, Yunnan Institute of Microbiology, School of Life Sciences, Yunnan University, Kunming 650091, People’s Republic of China

^2^ Zhaotong Health Vocational College, Zhaotong 657000, People’s Republic of China

^†^ These authors contributed equally to this work.

^*^ Correspondence: Xiao-Yang Zhi, [xyzhi@ynu.edu.cn](mailto:xyzhi@ynu.edu.cn)

**Supplementary tables**

All supplementary tables are organized in a Microsoft Excel file named supplementary_tables.xlsx.

**Supplementary Table 1.** The genome information of 403 type strains of genus *Streptomyces*. VP: Valid Publication; AL: Approved Lists 1980.

**Supplementary Table 2:** The detailed information of genomes removed from the basic genome dataset.

**Supplementary Table 3:** The grouping information of eight clades accommodated 373 *Streptomyces* genomes in the reference species tree.

**Supplementary Table 4:** The information of 100 representative genome datasets (RGDs). Each dataset contains 64 *Streevptomyces* genomes.

**Supplementary Table 5:** The average Robinson-Foulds (RF) distances of gene datasets compared to species trees based on supermatrix (ST1) and supertree (ST2), respectively.

**Supplementary Figures**

**Supplementary Figure 1. Reference species tree of *Streptomyces* based on 40 universal markers from 373 genomes.**

**_
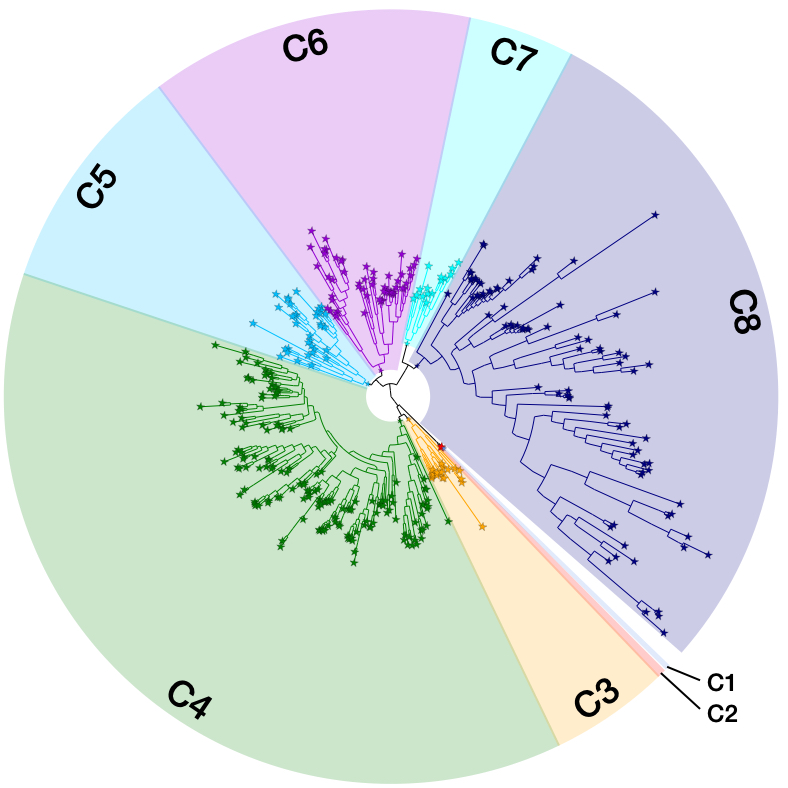
_**

**Supplementary Figure 2. Topological comparison between single-gene trees and species trees based on supertree (ST2).** Each single data point represented a mean value of Robison-Foulds distances between single-gene trees in the corresponding dataset (*e.g*., DG) to ST2 in one of 100 RGDs.

**Supplementary Figure 3. Robinson-Foulds distance of general gene tree and reconciled gene tree to ST2.** For a gene dataset, 100 mean values of RF distances corresponding to 100 RGDs were averaged and shown in the upper panel. Red solid circles represent the average RF distance of general gene trees to stST, and Blue solid circles represent the average RF distance of reconciled gene trees to stST. The relative RF distance was calculated as formula, $\frac{{\bar{\mathrm{RF}}}_{t_{J}\to ST2}-{\bar{\mathrm{RF}}}_{t_{\mathrm{sOG}}\to ST2}}{{\bar{\mathrm{RF}}}_{sOG\to ST2}}$, where $t_{J}$ is general gene tree of the gene in COG J. In the lower panel, arrows start from the relative RF distance of general gene trees and point to the relative RF distance of reconciled gene trees.

**Supplementary Figure 4. Regression analysis between RF distance (between the single-gene tree and ST2) and PIS numbers.**

**
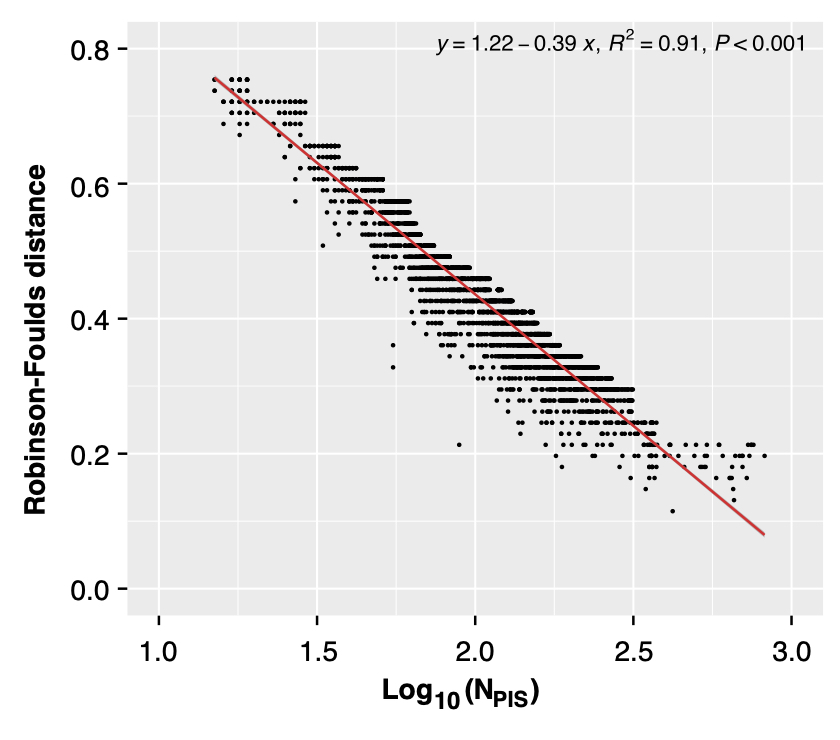
**

**Supplementary Figure 5. Normalized Robison-Foulds distances (upper panel) and relative normalized Robison-Foulds distances (lower panel) between single-gene trees and ST2.** Like the lower panel of Supplementary Figure 4, arrows start from the relative RF distances of general gene trees and point to the relative RF distances after normalization based on alignment length.
